# A review of the defining chemical properties of soda lakes and pans: An assessment on a large geographic scale of Eurasian inland saline surface waters

**DOI:** 10.1371/journal.pone.0202205

**Published:** 2018-08-20

**Authors:** Emil Boros, Marina Kolpakova

**Affiliations:** 1 Balaton Limnological Institute, Centre for Ecological Research, Hungarian Academy of Sciences (MTA), Tihany, Hungary; 2 Sobolev Institute of Geology and Mineralogy, Siberian Branch of Russian Academy of Sciences, Novosibirsk, Russia; 3 National Research Tomsk Polytechnic University, Tomsk, Russia; Fred Hutchinson Cancer Research Center, UNITED STATES

## Abstract

The aim of this study is to evaluate the definition of water chemical type, with particular attention to soda brine characteristics by assessing ionic composition and pH values on a large geographic scale and broad salinity (TDS) range of Eurasian inland saline surface waters, in order to rectify the considerable confusion about the exact chemical classification of soda lakes and pans. Data on pH and on the concentration of eight major ions were compiled into a database drawn from Austria, China, Hungary, Kazakhstan, Mongolia, Russia, Serbia, and Turkey. The classification was primarily based on dominant ions exceeding an equivalent percentage of 25 (> 25e%) of the total cations or anions, and the e% rank of dominant ions was also identified. We identified four major types: waters dominated by (1) Na-HCO_3_ (10.0%), (2) Na-HCO_3_ + CO_3_ (31.4%), (3) Na-Cl (45.9%), and (4) Na-SO_4_ (12.7%), considering only the first ion by e% rank. These major types can be divided into 30 subtypes in the dataset, taking into account the e% rank of all dominant ions. The major and subtypes of soda brine can be divided into “Soda” and “Soda-Saline” types. “Soda type” when Na^+^ and HCO_3_^–^ + CO_3_^2–^ are the first in the rank of dominant ions (> 25e%), and “Soda-Saline type” when Na^+^ is the first in the rank of dominant cations and the sum of HCO_3_^–^ + CO_3_^2–^ concentration exceeds 25e%, but it is not the first in the rank of dominant anions. Soda-saline type can be considered as a separate evolutionary stage between Soda and Saline types respect to the geochemical interpretation by saturation indexes of brines. The obtained overlapping ranges in distribution demonstrate that a pH measurement alone is not a reliable indicator to classify the permanent alkaline “soda type” and various other types of temporary alkaline waters.

## Introduction

Soda lakes and pans can be found on all continents, but they are much less widespread than other types of saline waters [1], and most of them are shallow with extreme physical and chemical conditions, special biogeochemical cycling and unique communities [2–3]. The combination of sodium and carbonates results in alkaline conditions, and such systems are termed as “soda lakes.” Brackish and saline alkaline lakes—“soda lakes”—have saline waters with sodium (Na^+^) and carbonate species (HCO_3_^–^ + CO_3_^2–^) as the dominant ions and typically exceed a pH of 9. Soda-lake waters also commonly have high concentrations of chloride, variable concentrations of sulfate and potassium, but they have very low concentrations of alkaline earths [4], because of the equilibrium state with carbonate minerals (calcite, high-magnesium calcite, strontianite, etc.). Since soda-lake formation depends on low levels of dissolved calcium and magnesium, as well as on the dominance of bicarbonate (HCO_3_^–^ >> Ca^2+^ + Mg^2+^), they represent the most stable high-pH environments (pH > 9) on Earth, which clearly distinguishes them from other inland saline waters [5–7]. The “Precambrian explosion” of prokaryote diversity might have taken place in alkaline environments [8], therefore, alkaline soda lakes are hypothesized to have been appropriate habitats of ancient prokaryotic communities [9], when the Archaean ocean was possibly dominated by Na-Cl-HCO_3_ and did not resemble the Na-Cl ocean of today [10]. The modern dilute soda lakes, particularly in tropical regions, are regarded as the most productive environments in the world, with primary producers having unlimited access to CO_2_ in the form of HCO_3_^–^ and CO_3_^2–^ [5,11]. Furthermore, soda lakes are fertile habitats for an enormous diversity of alkaliphilic prokaryotic microbiota, which in turn are hosts to a range of alkaliphilic viruses [12], and also for indigenous planktonic bacterial communities able to cope with the multiple extreme conditions present in unique soda pans [13]. Fundamentally, it can be concluded that the richness of HCO_3_^–^ and CO_3_^2–^, coupled with alkalinity, is the major chemical driver of soda lake and pan ecosystems.

The saline lake ecosystems of the world are important natural assets with considerable esthetic, cultural, economic, recreational, scientific, conservation, and ecological value [14]. This is particularly true for the unique trace element accumulation [15–16] and biodiversity [17–19] of soda lakes and pans. Although alkaline and soda lakes and pans are considered to be analogous expressions in many scientific fields, including limnology, the considerable variation in the proportions of bicarbonate (HCO_3_^–^), carbonate (CO_3_^2−^), chloride (Cl^–^), and sulfate (SO_4_^2–^) ions has led to the term “alkaline” often being used interchangeably with the term of “carbonate alkalinity” [20]. In the field of geochemistry, however, “alkaline” is associated with high pH values (pH > 7.0), while “alkalinity” is calculated as the sum of bicarbonates and carbonates in water (m_HCO3_^–^ + 2m_CO3_^2−^). The lack of precision in the use of these chemical terms can lead to the incorrect chemical identification of saline waters as soda brine, especially when pH is primarily taken into account.

Saline waters around the world are dominated by eight major solutes: Ca^2+^, Mg^2+^, Na^+^, K^+^, HCO_3_^–^, CO_3_^2–^, Cl^–^, and SO_4_^2–^, but several cation–anion incompatibilities exist in concentrated brines (high HCO_3_^–^ + CO_3_^2–^ concentrations are coupled with low Ca^2+^ and Mg^2+^ amounts; high SO_4_^2–^ concentrations are combined with low Ca^2+^; Ca^2+^ as a dominant ion usually correlates with high Cl^−^concentrations). Therefore, the number of major brine types is limited [21], but the chemical types of lakes are differently classified, and probably no classification is entirely satisfactory, especially not in the case of soda lakes and pans.

Most authors distinguish four or five major saline water types using a minimum of 25 mol or equivalent percentage threshold for ions. These major types can be subdivided to give several subtypes based on the rank of major ions with an equivalent percentage exceeding 25% [1, 21–22]. Although there is an evidence of major soda brine used by limnologists (Na-HCO_3_-CO_3_), various combinations of ions exist for the classification of soda brine, which are described in many comprehensive papers and citations, such as Na-CO_3_, Na-HCO_3_-CO_3_, Na-CO_3_-Cl-SO_4_, and Na-HCO_3_-CO_3_-Cl. In this case, the predominance of Na as the most dominant cation is sufficient, but such a simple criterion is misleading, because sodium is by far the most common dominant cation in saline lakes around the world [1]. The Kurlov equation, which includes total dissolved solids (TDS), pH, and the equivalent percentage of ions, can be used for the visual interpretation of ion composition [23].

In Russia, a method developed by MG Valyashko [24–25] is used for the classification of inland reservoir waters. According to this method, the so-called “metamorphic coefficients” (*K*_1_, *K*_2_, *K*_3_, and *K*_4_, shown in Table 1) are used to determine the chemical type, which can be carbonate, sulfate (sodium or magnesium) or chloride (see Table 1). First, index *K*_1_ (or “soda index”, as referred to hereinafter) is used as an indicator of carbonate type, the second and third indices are used for the identification of sulfate and chloride types, and the last one (fourth) divides sulfate type into two subtypes: sodium and magnesium sulfate. In China, Zheng [26] further subdivided the carbonate type of that classification into strong, moderate, and weak carbonate subtypes based on the ratio (*K*_c_) of the weight percentage of carbonate plus bicarbonate and the total amount of salts.

**Table 1:** Main features of “metamorphic coefficients” in different types of inland saline waters according to Valyashko [25].

Chemical type	Subtype	Metamorphic coefficients			
		*K*_1_	*K*_2_	*K*_3_	*K*_4_
**Carbonate (soda)**	−	> 1	>> 1	>> 1	>> 1
**Sulfate**	Sodium sulfate	≤ 1	≥ 1	>> 1	≠ 1
**Sulfate**	Magnesium sulfate	<< 1	≤ 1	>> 1	≠ 1
**Chloride**	−	<< 1	<< 1	≤≤ 1	< 1

Footnotes

Where $K_{1}=\frac{C({CO}_{3}^{2-})+C({HCO}_{3}^{-})}{{C(Ca}^{2+})+C{(Mg}^{2+)}};K_{2}=\frac{C({CO}_{3}^{2-})+C({HCO}_{3}^{-})+C({SO}_{4}^{2-})}{{C(Ca}^{2+})+C{(Mg}^{2+)}};K_{3}=\frac{C({CO}_{3}^{2-})+C({HCO}_{3}^{-})+C({SO}_{4}^{2-})}{{C(Ca}^{2+})};K_{4}=\frac{C({CO}_{3}^{2-})+C({HCO}_{3}^{-})}{{C(Ca}^{2+})}$

In the context of the classifications and terms described above, the overall aim of this study is to evaluate the definition of water chemical types, with regard to the soda brine character in particular by assessing ion composition and pH over a large geographic scale (along a 7000-km-long gradient in eight countries) and a broad salinity (TDS) range (brine, saline, and brackish) of Eurasian saline waters. Due to the lack of exact chemical definition for soda lakes and pans, two hypotheses are explored in the study:

(1) The dominance and priority of Na^+^ and HCO_3_^–^ + CO_3_^2–^ ions can precisely define the soda brine character of waters if dominance means a minimum of 25 equivalent percentage (e%) and priority by the rank of dominant ions. (2) The pH of soda lakes is significantly higher and has a distinct range compared to other types of saline waters.

## Materials and methods

### Study sites

**Fig 1:**
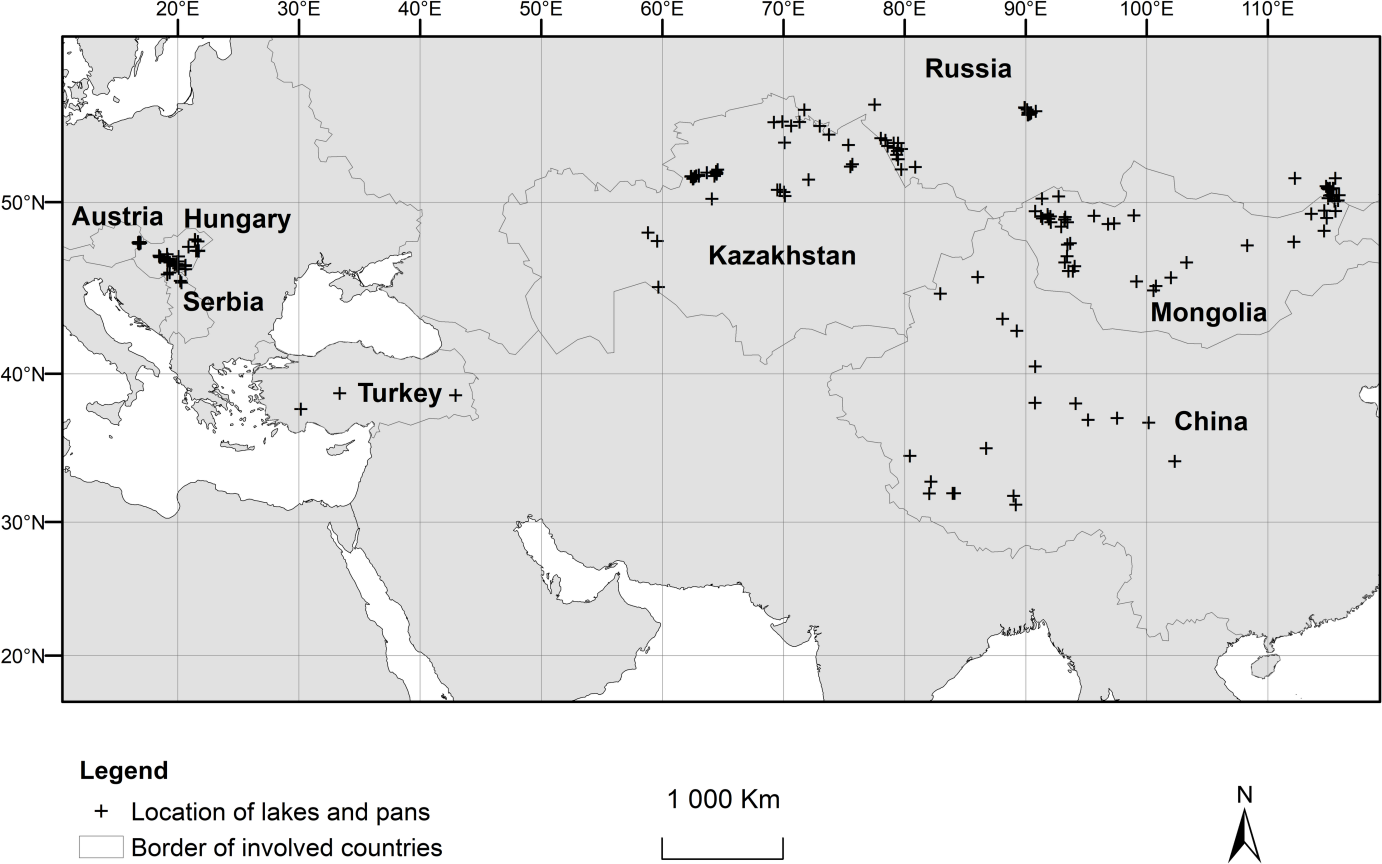
The large geographic scale distribution of the assessed lakes and pans in the dataset by countries and references. Footnotes Austria, Hungary, Serbia (*N* = 72) [27–28]. China (*N* = 20) [26,29]. Kazakhstan (*N* = 30) [30–32] and in S1 Table (*N* = 7). Mongolia (*N* = 26) [15] and in S1 Table (*N* = 12). Russia (*N* = 50) [33–39]. Turkey (*N* = 3) [1].

A variety of different surface inland saline waters was assessed in our study, including all types of lakes, pans, wetlands, and inland saline water systems not directly connected to an ocean. These inland saline water systems were selected from arid and semi-arid zones of different geographic regions of Eurasia (Anatolia, Carpathian Basin, Central Asia, Inner Mongolia, Eastern and Western Siberia, and Tibet), where major ion composition data from soda lakes and pans was presumably available. Because of limited data availability from several potential regions, the presented information is not a complete survey of soda lakes and pans in Eurasia, but it does provide an overview of the soda chemical types of inland saline systems in the area. The chemical data came from original articles and the selection was not dependent on the classification of saline waters, on hydrogeological origin, attributes, water regime, depth, or size (above 1 ha). The locations of the sites were processed by QGIS 2.18 Las Palmas de G.C. software based on Google Satellite map, and the distribution map (Fig 1) was built using ESRI ArcMap 10.2 GIS software.

### Data collection and measurements

Major ion concentration data including sodium (Na^+^), potassium (K^+^), calcium (Ca^2+^), magnesium (Mg^2+^), chloride (Cl^–^), sulfate (SO_4_^2–^), bicarbonate (HCO_3_^–^), carbonate (CO_3_^2–^), and pH (if it was coupled with ion data) were compiled into a database. The data were drawn from a large geographic scale across Eurasia (Austria, China, Hungary, Kazakhstan, Mongolia, Russia, Serbia, and Turkey) and from a large number of saline lakes and pans (*N* = 220) with a minimum of 1.0 g L^–1^ salinity threshold. The 1.0 g L^–1^ salinity threshold was selected on the basis of a former study [27], where this threshold was experimentally found to be the characteristic boundary of soda ecosystems. Salinity (TDS) was estimated by the sum of major ion concentrations (Na^+^, K^+^, Ca^2+^, Mg^2+^, Cl^–^, SO_4_^2–^, HCO_3_^–^, and CO_3_^2–^) and 1 mg L^-1^ accuracy was used for all input concentration data independently of the theoretical accuracy of original measurements, therefore decimals were rounded to integer value. As generally known, sodium is by far the most common cation in saline lakes and necessarily the dominant cation in soda type of lakes [1]. Therefore, sites with the most abundant ion other than Na^+^ were excluded from the dataset. If seasonal or annual water data were available, we used mean values for the database. Most of the data came from papers [1,15,26–39] published in the period from 1956 to 2017. However, we cannot estimate a reliable universal accuracy and reproducibility for all data in this study, because none of the published papers reviewed provide data on uncertainty, furthermore, several of them were carried out by different kinds of national standard methods of ion concentration measurements, but all of them are based on the same international methods of ICP-MS for cations (ASTM 3500-Na-C, -K-C, -Ca-C, -Mg-C), alkalinity titration (ASTM 2320 B), chloride argentometric titrimetry (ASTM 4500-Cl^-^B) as well as sulfate ions turbidimetry (ASTM 4500-SO_4_^2-^E). The large geographic distribution of the lakes and pans assessed in the dataset can be seen in the Fig 1 by countries and references.

The collected published data were supplemented with some unpublished sample series in the case of Mongolia and Kazakhstan. Coordinates, countries, sites, pH, and ion composition (e%) data of these additional sites are listed in S1 Table. pH was measured on site using a WTW multiline field instrument with a SenTix 41 electrode. The samples were filtered through GF5 glass fiber filters (0.4 μm nominal pore size) in the laboratory. For the determination of cations (Na^+^, K^+^, Ca^2+^, and Mg^2+^), samples were analyzed with the EPA 6020 standard method of inductively coupled plasma mass spectrometry [40] with 2% accuracy. Anion concentrations were measured according to Hungarian standard analytical methods (MSZ). Chloride ion concentration was determined by the argentometric method (MSZ 448–15:1982). Sulfate ion was precipitated in a strong acidic medium with barium chloride, and the resulting turbidity was measured photometrically at 405 nm and compared with standard solutions (MSZ 12750–16:1998). Alkalinity was determined by titration, and the concentration of HCO_3_^–^, CO_3_^2–^, and OH^−^ions was calculated using the Hungarian standard MSZ 448–11:1986. All of anions and alkalinity were measured with 5% accuracy.

### Analysis of data

Classification was developed using the concentration of dominant ions with 1 mg precision, as indicated by values exceeding 25 equivalent percentage (> 25e%) of the total amount of cations or anions. The equivalent percentage of each ion was calculated separately for cations (1) and anions (2):

$${e\%}^{\left(i\right)}\mathrm{cat}=\left[\frac{\frac{C_{i}}{{M_{m}}^{-F_{e}}}}{\sum_{4}cat(\frac{C_{i}}{{M_{m}}^{-F_{e}}})}\right]\times 100,$$ (1)

$${e\%}^{\left(i\right)}\mathrm{ani}=\left[\frac{\frac{C_{i}}{{M_{m}}^{-F_{e}}}}{\sum_{4}ani(\frac{C_{i}}{{M_{m}}^{-F_{e}}})}\right]\times 100,$$ (2)

where

e%^(i)^_cat_ = equivalent percentage of one (i) cation.

e%^(i)^_ani_ = equivalent percentage of one (i) anion.

*C*_i_ = ion concentration (mg L^–1^).

*M*_m_ = molar mass.

*F*_e_ = equivalence factor, values are 1 (if ion charge ±1) or 2 (if ion charge ±2).

The sequence of dominant ions was identified separately for cations and anions [1,21]. Carbonate and bicarbonate ions were treated as a sum (HCO_3_^–^ + CO_3_^2–^), because the dissociation relationship and the proportions of CO_2_, HCO_3_^–^ and CO_3_^2–^ significantly depend on pH, and carbonate ion occurs in adequate amount if pH exceeds 8.4 [20]. In addition, a “soda index” (3) was applied to identify soda brine character (Table 1) [25]:

$$\frac{C({\mathrm{HCO}}_{3}{}^{-})+C({\mathrm{CO}}_{3}{}^{2-})}{C({\mathrm{Ca}}^{2+})+C({\mathrm{Mg}}^{2+})}>1,$$ (3)

where C = equivalent mass form of ions (gram equivalent).

The *K*_c_ value (*K*_c_ = (Na_2_CO_3_ + NaHCO_3_ / total salt) × 100, where amounts are expressed as weight percent) used by Zheng et al. [26] to subdivide the carbonate type into strong, moderate, and weak carbonate subtypes was not calculated, since the unit of measurement for the concentration data included in the current assessment was g L^−1^ –an up-to-date, conventional unit in limnology and geochemistry, which is not precisely interchangeable with mass or density data [22].

The PCA ordination of ion composition data was performed using OriginPro 9 (OriginLab, Northampton, MA) software with a significance level of *p* < 0.05. Kruskal–Wallis test and Dunn test were used for salinity and pH comparison. The overlapping coefficient for pH, as a measure of coincidence between normal probability distributions, was calculated in R [41] using the method of Inman and Bradley [42].

The thermodynamic calculations were performed assuming 25°C and 1 atm total pressure using the PHREEQC 3.1.4 [43] and PITZER database. The program algorithm is based on the equations of the mass action law and knowledge of the solubility and dissociation constants. The main reason for the software choice was a possibility of using the method of Pitzer [44] designed for highly mineralized waters and brines.

## Results

The salinity of the examined lakes varied across a broad range (1 g L^−1^ up to 390 g L^−1^), so did ion composition. Based on the calculations, ion composition can be divided into major types and subtypes based on the rank of the dominant ions (e%), with cations and anions treated separately. Four major types were found in the reviewed dataset: (1) Na-HCO_3_ (10.0%), (2) Na-HCO_3_ + CO_3_ (31.4%), (3) Na-Cl (45.9%), and (4) Na-SO_4_ (12.7%), considering only the first dominant ions by equivalent percentage (> 25e%) across the cation and anion pool. The major types can be further divided into 30 subtypes by taking into account the e% rank of all dominant ions (> 25e%). The most frequent subtypes were Na-Cl (15.9%), Na-HCO_3_-CO_3_ (13.2%), Na-HCO_3_ (6.8%), Na-Cl > SO_4_ (9.1%), Na-Cl > HCO_3_-CO_3_ (8.2%), Na-HCO_3_-CO_3_ > Cl (7.3%), and Na > Mg-Cl (6.4%). The contribution of all other ion combinations in the dataset was under 5% (Table 2). As can be seen, the relative occurrence of the four major types noted above significantly decreases after subdividing each type, and in some cases, the percentage is reduced by half.

**Table 2:** The chemical types of lakes and pans with groups of major and subtypes identified on the basis of the dominant ion method.

Major type	Subtype	*N*	%	Chemical types
**Na-HCO**_**3**_	Na-HCO_3_	15	6.8	Soda
**Na-HCO**_**3**_	Na-HCO_3_ > Cl	4	1.8	Soda
**Na-HCO**_**3**_	Na-HCO_3_ >Cl > SO_4_	1	0.5	Soda
**Na-HCO**_**3**_	Na > Mg-HCO_3_	1	0.5	Soda
**Na-HCO**_**3**_	Na > Mg-HCO_3_ > Cl	1	0.5	Soda
*Total of major type*		*22*	*10*,*0*	
**Na-HCO**_**3**_**-CO**_**3**_	Na-HCO_3_-CO_3_	29	13.2	Soda
**Na-HCO**_**3**_**-CO**_**3**_	Na-HCO_3_-CO_3_ > Cl	16	7.3	Soda
**Na-HCO**_**3**_**-CO**_**3**_	Na-HCO_3_-CO_3_ > SO_4_	9	4.1	Soda
**Na-HCO**_**3**_**-CO**_**3**_	Na-HCO_3_-CO_3_ > SO_4_ > Cl	1	0.5	Soda
**Na-HCO**_**3**_**-CO**_**3**_	Na > Ca-HCO_3_-CO_3_	1	0.5	Soda
**Na-HCO**_**3**_**-CO**_**3**_	Na > Mg-HCO_3_-CO_3_	6	2.7	Soda
**Na-HCO**_**3**_**-CO**_**3**_	Na > Mg-HCO_3_-CO_3_ > Cl > SO_4_	2	0.9	Soda
**Na-HCO**_**3**_**-CO**_**3**_	Na > Mg-HCO_3_-CO_3_ > SO_4_	5	2.3	Soda
*Total of major type*		*69*	*31*,*4*	
**Na-Cl**	Na-Cl	35	15.9	Saline
**Na-Cl**	Na-Cl > HCO_3_	2	0.9	Soda-Saline
**Na-Cl**	Na-Cl > HCO_3_-CO_3_	18	8.2	Soda-Saline
**Na-Cl**	Na-Cl > HCO_3_-CO_3_ > SO_4_	2	0.9	Soda-Saline
**Na-Cl**	Na-Cl > SO_4_	20	9.1	Saline
**Na-Cl**	Na-Cl > SO_4_ > HCO_3_-CO_3_	2	0.9	Soda-Saline
**Na-Cl**	Na > Mg-Cl	14	6.4	Saline
**Na-Cl**	Na > Mg-Cl>SO_4_	8	3.6	Saline
*Total of major type*		*101*	*45*,*9*	
**Na-SO**_**4**_	Na-SO_4_	3	1.4	Saline
**Na-SO**_**4**_	Na-SO_4_ > Cl	10	4.5	Saline
**Na-SO**_**4**_	Na-SO_4_ > HCO_3_-CO_3_	3	1.4	Soda-Saline
**Na-SO**_**4**_	Na-SO_4_ > HCO_3_-CO_3_ > Cl	2	0.9	Soda-Saline
**Na-SO**_**4**_	Na > Mg-SO_4_	3	1.4	Saline
**Na-SO**_**4**_	Na > Mg-SO_4_>Cl	4	1.8	Saline
**Na-SO**_**4**_	Na > Mg-SO_4_ > Cl > HCO_3_-CO_3_	1	0.5	Soda-Saline
**Na-SO**_**4**_	Na > Mg-SO_4_ > HCO_3_-CO_3_	1	0.5	Soda-Saline
**Na-SO**_**4**_	Na > Mg-SO_4_ > HCO_3_-CO_3_ > Cl	1	0.5	Soda-Saline
*Total* ***of major types***		*28*	*12*,*7*	
**Total**		220	100.0	

Footnotes

Major types: identified only by the most dominant cation and anion (> 25e%).

Subtypes: identified by the rank of dominant ions (> 25e%).

The term “saline” here means all the other types of inland saline waters without soda brine character.

Focusing on the soda brine characteristic across the diverse series of ion composition, there were 91 sites (41%) where the e% of HCO_3_^–^ + CO_3_^2–^ was the first in the rank of anions with more than 25e%. Thus, this group was classified as characteristically “Soda” chemical type. Another group of 32 sites (15%) was found to be an intermediate between Soda and Saline types, where HCO_3_^–^ + CO_3_^2–^ exceeded 25e%, but were not of the most dominant anions, therefore these sites were classified as “Soda-Saline” chemical type. The third group of “Saline” waters had weak or no soda brine characteristic and consisted of 97 sites (44%), where HCO_3_^–^ + CO_3_^2–^ content was less than 25e%. According to the rank of dominant ions, the soda brine-associated types and subtypes can be divided into two major groups as follows (Table 2):

Soda group:

- Na^+^ > 25e% and first in the rank of dominant cations (Na^+^ > than any other cation e%)
- HCO_3_^–^ + CO_3_^2–^ > 25e% and first in the rank of dominant anions (HCO_3_^–^ + CO_3_^2–^ > than any other anion e%)

Soda-Saline group:

- Na^+^ > 25e% and first in the rank of dominant cations (Na^+^ > than any other cation e%)
- HCO_3_^–^ + CO_3_^2–^ > 25e% but not the most dominant anions (HCO_3_^–^ + CO_3_^2–^ < than Cl^−^or SO_4_^2–^ e%)

**Fig 2:**
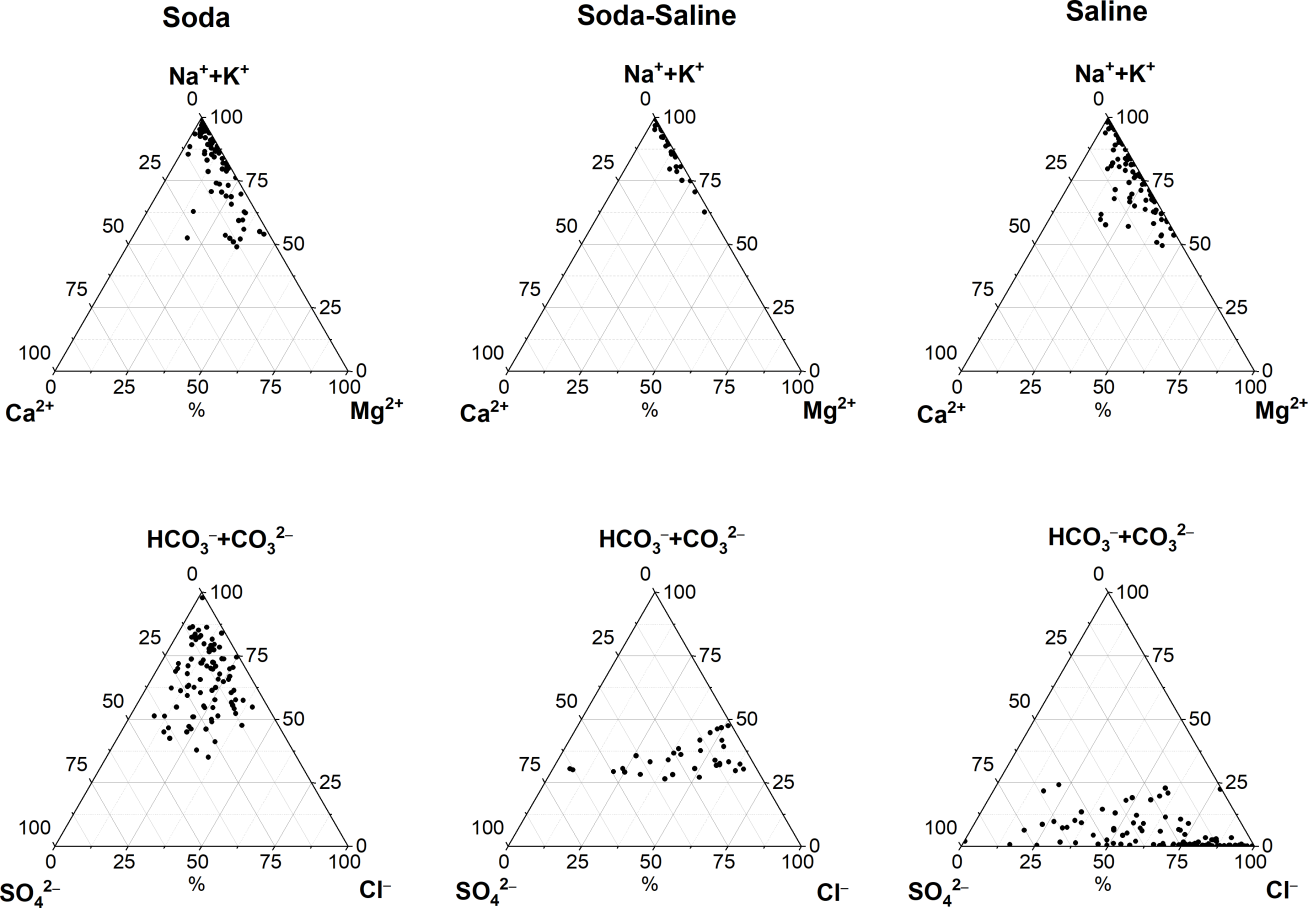
Ternary diagrams of the equivalent percentage (e%) contribution of ions to total ion content in the identified water chemical types. Footnotes The term “saline” here means all the other types of inland saline waters without soda brine character.

The Soda brine group contains 13 subtypes of different ion composition with Mg^2+^, Cl^−^and SO_4_^2–^ being the most frequent dominant ions besides Na^+^, HCO_3_^–^, and HCO_3_^–^ + CO_3_^2–^ (Ca^2+^ ion occurred only in one lake). The Soda-Saline group is composed of 9 subtypes with varying ion combination including Mg^2+^, Cl^–^, SO_4_^2–^ and HCO_3_^–^ + CO_3_^2–^ ions. Ca^2+^ ion did not occur in this group. The equivalent percentage contribution of an ion to total ion content of the major and subtype groups is summarized in the ternary graphs of Fig 2.

The geographic distribution of the identified types was quite heterogeneous across the sampled regions. Characteristic soda type of lakes and pans occurred in the largest numbers in the Carpathian Basin (68%; Austria, Hungary, and Serbia), followed by Russia (14%) and Mongolia (13%). The occurrence of Soda-Saline type waters was highest in Russia and Mongolia (44% and 31%, respectively), where the share of all the other saline types (16% and 24%, respectively) was similar to that of the Soda type. This third group of waters can be mostly found in China and Kazakhstan (Table 3).

**Table 3:** Number of lakes and pans by identified chemical type and country.

Country	Soda	Soda-Saline	Saline	Sum
**Austria**	30	2		32
**China**			20	20
**Hungary**	32	4	1	37
**Kazakhstan**	2		35	37
**Mongolia**	12	10	16	38
**Russia**	13	14	23	50
**Serbia**	2	1		3
**Turkey**		1	2	3
***Sum***	*91*	*32*	*97*	*220*

Footnotes

The term “saline” here means all the other types of inland saline waters without soda brine character.

**Fig 3:**
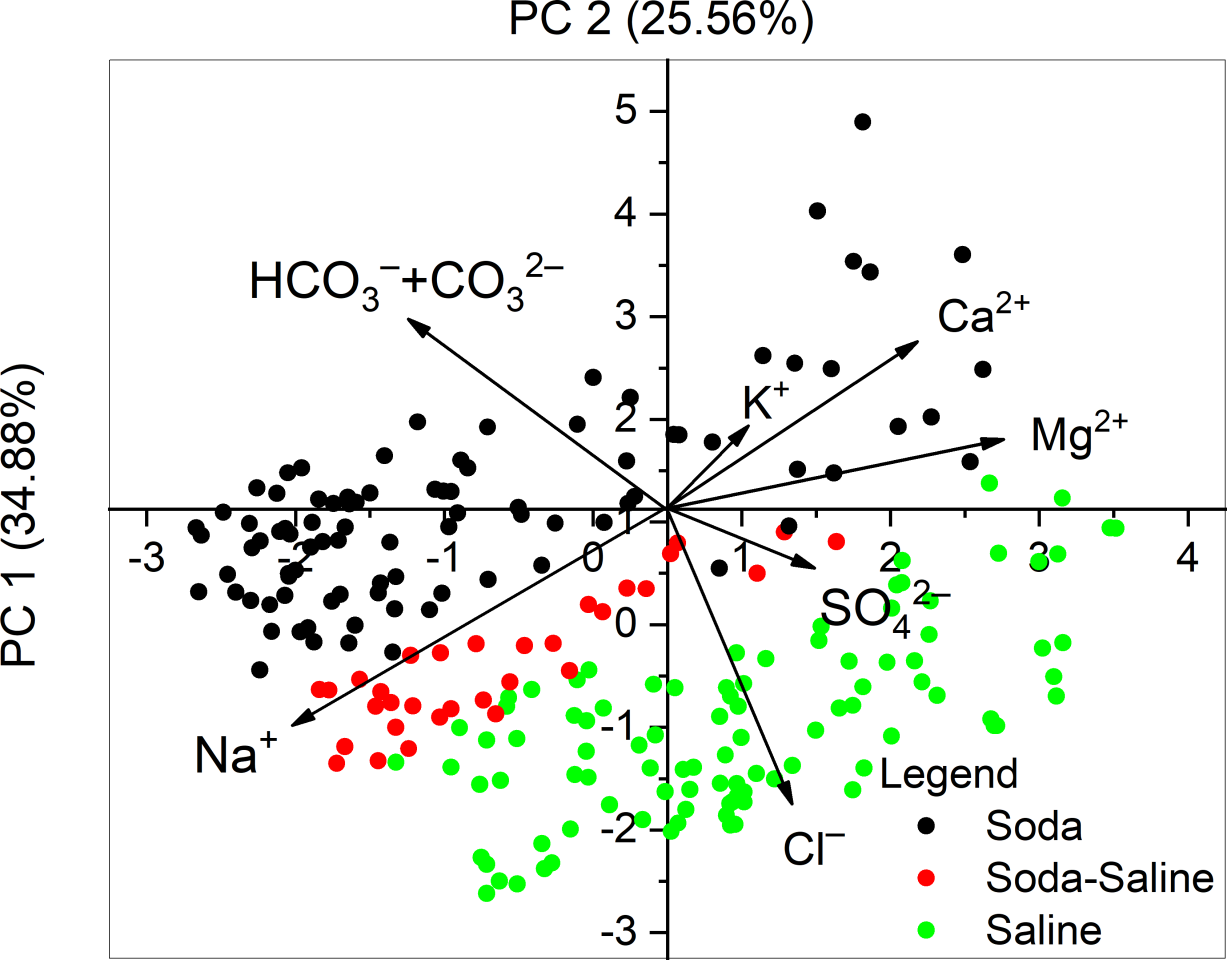
Principal component analysis (PCA) performed on the ion composition of the identified water chemical types. Footnotes The term “saline” here means all the other types of inland saline waters without soda brine character.

**Fig 4:**
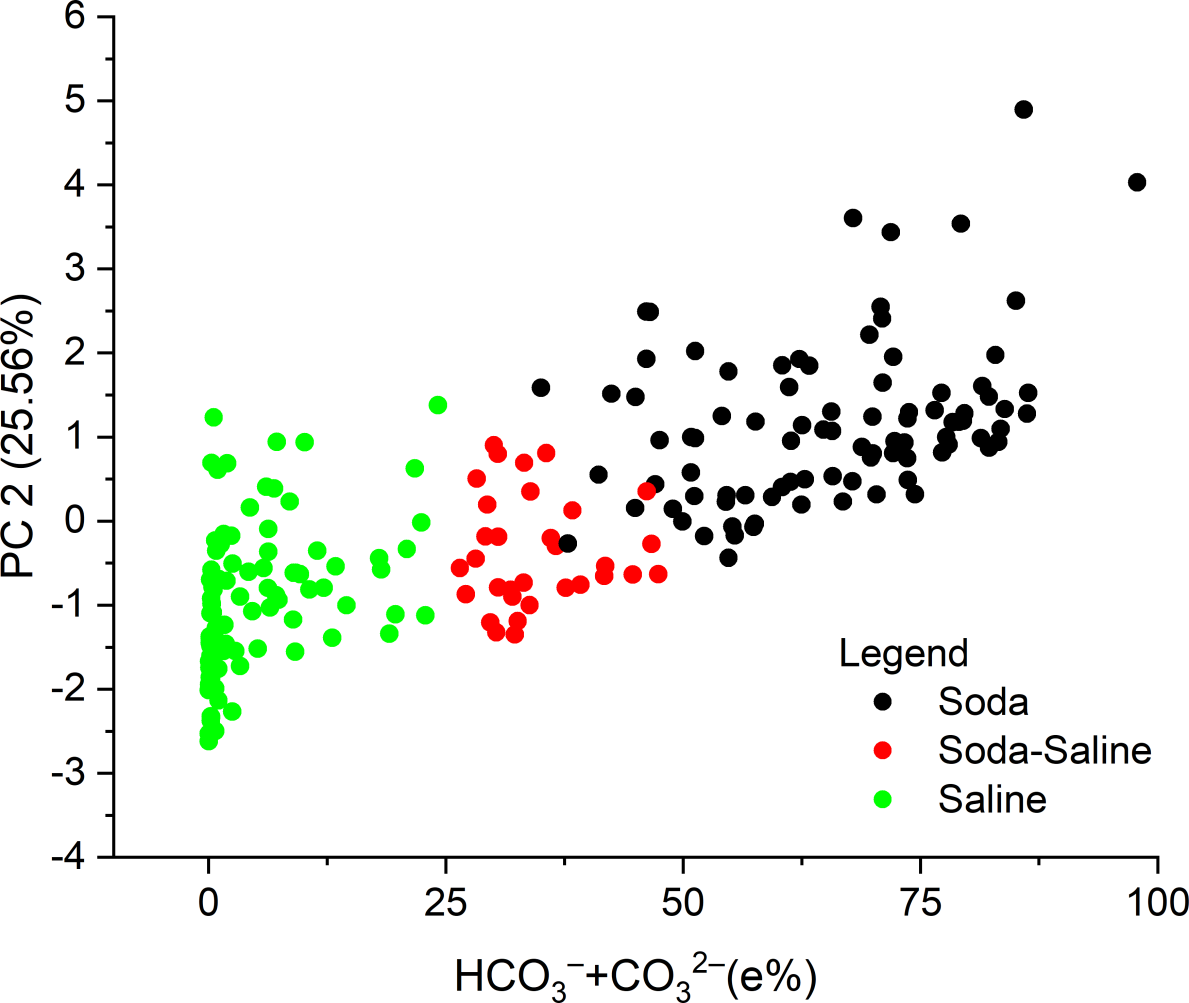
Relationship between the equivalent percentage (e%) of HCO_3_^–^ + CO_3_^2–^ and its loadings (scores) in the PCA with the identified water chemical types. Footnotes The term “saline” here means all the other types of inland saline waters without soda brine character.

According to the PCA (Table 4 and Fig 3), the equivalent percentage of Na^+^ and HCO_3_^–^ + CO_3_^2–^ negatively correlated with the rest of the ions along the first axis, which explained 34.88% of total variation. The equivalent percentage of Na^+^, Cl^–^, and SO_4_^2–^ also negatively correlated with other ions along the second axis, which explained 25.56% of the variation. The site scores along the two PC axis show a clear separation of the Soda, Soda-Saline and Saline groups. HCO_3_^–^ + CO_3_^2–^ ions, as the key elements of the classification scheme, positively correlated to the second PC axis, along which a strict separation of the chemical types can be observed with increasing percentages of HCO_3_^–^ + CO_3_^2–^ (Fig 4).

**Table 4:** Loadings (scores) of the variables (ions) on the first two axes of the PCA ordination (Fig 3).

	PC1	PC2
**Na**^**+**^	−0.579	−0.284
**K**^**+**^	0.078	0.149
**Ca**^**2+**^	0.281	0.412
**Mg**^**2+**^	0.562	0.153
**Cl**^**–**^	0.252	−0.607
**SO**_**4**_^**2–**^	0.244	−0.032
**HCO**_**3**_^**–**^ **+ CO**_**3**_^**2–**^	−0.375	0.579

Footnotes

The term “saline” here means all the other types of inland saline waters without soda brine character.

However, there was no correlation (*r*^2^ = 0.080) between the percentage of HCO_3_^–^ + CO_3_^2–^ (> 25e%) and the Valyashko index values, and the resulting overlap was 79% (173 sites) between the two classification methods (Table 5). It is important to note that all Soda and Soda-Saline types of brines, and even 14 saline type waters, would have been identified as Soda type (or carbonate type, see Table 1) by the Valyashko method.

**Table 5:** Number and ion composition of different chemical types of lakes and pans by dominant ions and by Valyashko classification, and the amount of overlap between the two methods.

Chemical type	*N*	Na^+^	K^+^	Ca^2+^	Mg^2+^	Cl^–^	SO_4_^2–^	HCO_3_^–^ + CO_3_^2–^	HCO_3_^–^	CO_3_^2–^
		e%				e%				
**Types by dominant ion**										
**Soda**	91	45–99	0–8	0–29	0–44	0–80	1–98	2–98	1–86	0–97
**Soda-Saline**	32	62–98	62–98	0–6	0–5	6–65	1–64	27–47	11–47	0–22
**Saline**	96	49–96	0–15	0–23	1–46	16–98	1–83	0–24	0–22	0–7
**Types by Valyashko index**										
**Soda**	136	45–99	0–13	0–29	0–44	0–80	1–98	2–98	1–86	0–97
**Sulfate and chloride**	83	49–96	0–15	0–23	1–46	16–99	0–83	0–35	0–33	0–7
**Intersection of Soda type by Valyashko index and dominant ion classification types**										
**Soda**	90	45–99	45–99	0–6	0–29	1–40	1–40	41–98	1–86	0–97
**Soda-Saline**	32	62–98	62–98	0–6	0–5	6–65	1–64	27–47	11–47	0–22
**Saline**	14	85–99	85–99	0–13	0–4	18–80	18–98	2–23	1–21	1–16

Footnotes

The term “saline” here means all the other types of inland saline waters without soda brine character.

**Fig 5:**
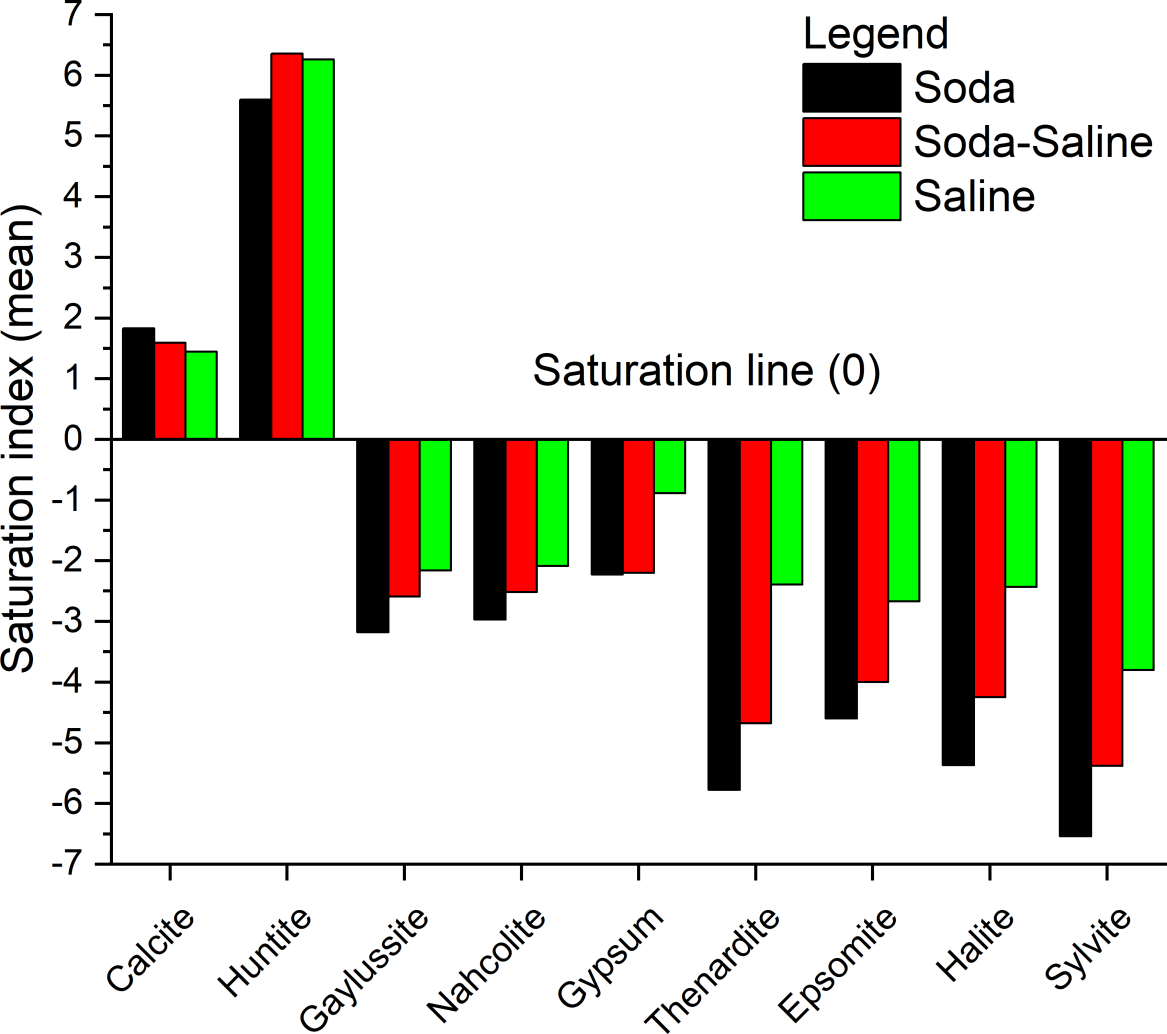
Mean values of the saturation index of Soda, Soda-Saline and Saline water chemical types with respect to major mineral assemblages that appear during the evaporation path.

The thermodynamic calculations showed that the three groups described are different in their equilibrium state with regard to main minerals that occur during the evaporation process of the solutions. Fig 5 shows that all of the studied waters are in oversaturated state with respect to calcium and magnesium carbonates (Calcite and Huntite) indicating that all of them have passed the tipping point of Calcite saturation and already contain sodium carbonate. Soda and Soda-Saline types are the less saturated with respect to sulfate and chloride minerals (Thenardite, Epsomite, Halite) and never reach the equilibrium point with them (Table 6). However, as can be seen from Fig 5 and Table 6, the Soda-Saline type is closer to the saturation line for these minerals in comparison with the Soda type. The Saline type is the most saturated with respect to the majority of minerals (Table 6) since their chemical composition is more enriched by major ions except for precipitated calcium and carbonate ions. The obtained results imply that the Soda-Saline type can be considered as a following Soda type stage of chemical evolution under conditions of high evaporation level.

**Table 6:** Mean, median, minimum and maximum values of the saturation index of Soda, Soda-Saline and Saline chemical types of waters with respect to major mineral assemblages that appear during the evaporation path.

Mineral name	Soda				Soda-Saline				Saline			
	Mean	Median	Min	Max	Mean	Median	Min	Max	Mean	Median	Min	Max
Calcite CaCO_3_	1,83	1,90	-0,27	3,06	1,59	1,55	0,44	2,74	1,44	1,33	-1,77	4,01
Huntite Mg_3_Ca(CO_3_)_4_	5,60	5,91	-2,49	8,83	6,36	6,50	0,92	10,4	6,26	6,30	-4,91	13,0
Gaylussite Na_2_Ca(CO_3_)_2_5H_2_O	-3,18	-2,94	-7,58	0,52	-2,59	-2,58	-5,29	0,61	-2,16	-2,07	-8,21	2,81
Nahcolite NaHCO_3_	-2,97	-3,05	-4,21	-1,15	-2,52	-2,63	-3,57	-0,81	-2,09	-2,18	-4,07	0,18
Gypsum CaSO_4_· 2H_2_O	-2,23	-2,26	-3,44	-1,10	-2,20	-2,16	-3,48	-0,96	-0,89	-0,87	-3,04	1,10
Thenardite Na_2_SO_4_	-5,78	-5,79	-7,98	-3,34	-4,68	-4,82	-6,99	-2,18	-2,39	-2,30	-7,58	0,89
Epsomite MgSO_4_·7H_2_O	-4,60	-4,60	-7,09	-3,30	-4,00	-3,93	-6,37	-2,95	-2,67	-2,67	-5,38	-0,41
Halite NaCl	-5,37	-5,53	-6,85	-2,93	-4,25	-4,32	-6,22	-2,39	-2,43	-2,55	-5,71	1,17
Sylvite KCl	-6,54	-6,64	-7,84	-4,78	-5,38	-5,38	-7,15	-2,96	-3,80	-3,86	-6,74	-0,42

**Fig 6:**
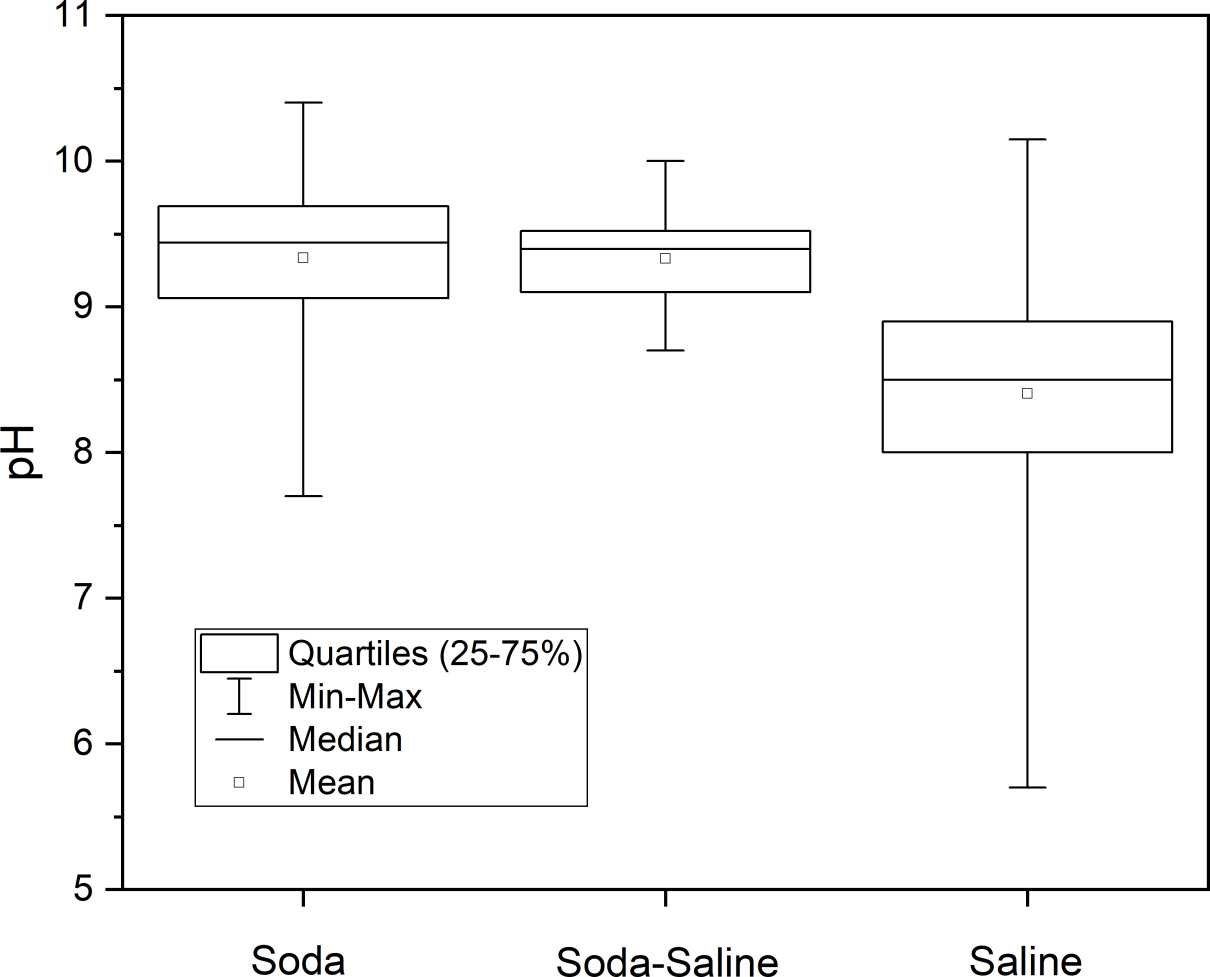
Means, medians, ranges (min−max), and quartiles (25%−75%) of pH in the identified water chemical types. Footnotes The term “saline” here means all the other types of inland saline waters without soda brine character.

On the basis of the Kruskal–Wallis and Dunn tests, we detected significant differences among the median salinity scores and pH values of the three identified chemical types (*N*_Soda_ = 91; *N*_Soda-Saline_ = 32; *N*_Saline_ = 97; *χ*^2^ = 111.334; df = 2; *p* >> 0.0005 for salinity and *N*_Soda_ = 91; *N*_Soda-Saline_ = 32; *N*_Saline_ = 84; *χ*^2^ = 71.080; df = 2; *p* >> 0.0005 for pH values). The highest mean (108 g L^−1^), median (70 g L^−1^) and maximum (390 g L^−1^) salinity values were found in Saline-type waters, while the values of the Soda-Saline (mean: 11 g L^−1^, median: 6 g L^−1^, and maximum: 70 L^−1^) and Soda (mean: 5 g L^−1^, median: 3 g L^−1^, and maximum: 45 L^−1^) chemical types had an order of magnitude lower salinity than Saline types, but the Soda-Saline types had also significantly higher salinity than Soda types. Mean pH in Soda and Soda-Saline brines was the same (9.33), and the median values were also quite similar (9.44 and 9.40), with no significant differences detected indicating that even a small amount of sodium carbonate in the solution creates a highly alkaline environment. By contrast, the mean (8.40) and median (8.50) pH of Saline type waters with negligible carbonate content were significantly lower than those of the other two groups (Fig 6).

**Fig 7:**
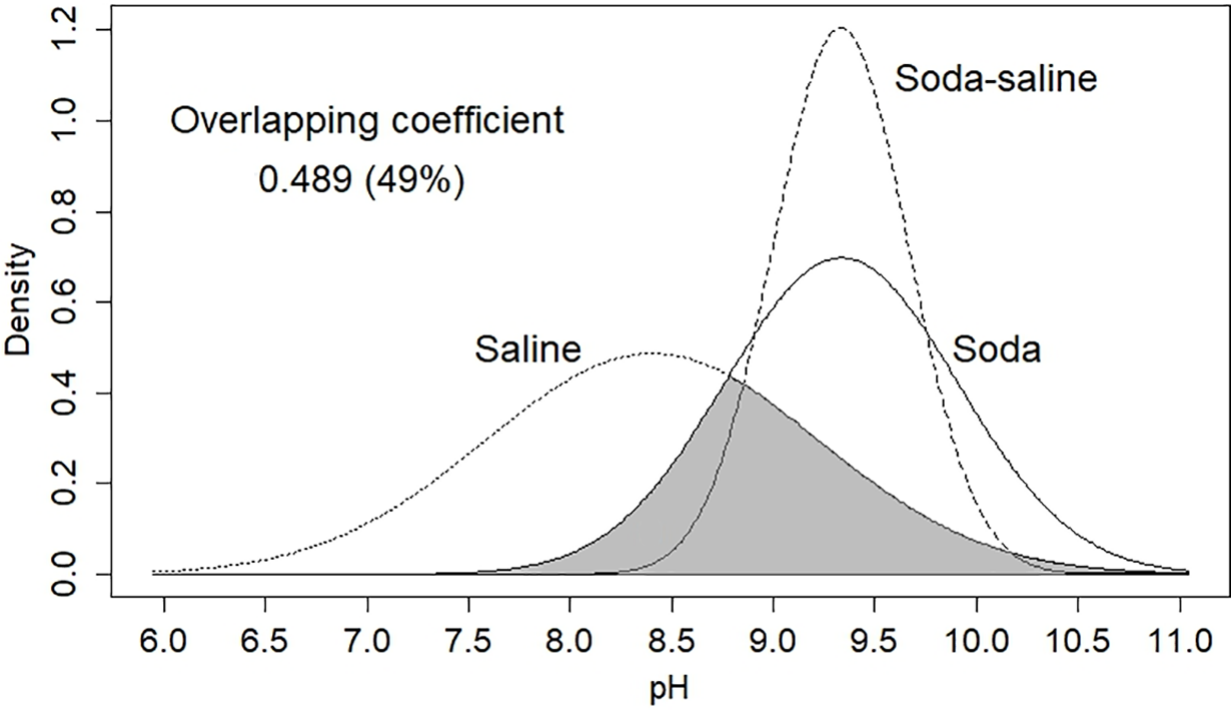
Overlap of the pH probability distribution between the identified water chemical types. Footnotes Gray color represents the total overlap area of the density distributions. The term “saline” here means all the other types of inland saline waters without soda brine character.

The range of salinity (2–390 g L^−1^) and pH (5.70–10.15) was the largest for Saline type waters. The range of salinity was similar in the Soda and Soda-Saline types, while the pH range was much narrower for the Soda (7.70−10.40) than the Saline types, and the narrowest for the Soda-Saline type (8.70−10.00). Despite the significant differences (Fig 6), pH values show that there is a considerable overlap in range (7.5−10.5) between Soda and Saline types in the pH probability distribution density (Fig 7), with a 0.489 (49%) total overlapping coefficient (see the gray color area in Fig 7) of all chemical types. Besides, the correlation (*r*^2^ = 0.330) between Salinity and pH was not significant.

## Discussion

Hammer [1] divided athalassic saline waters into three types based on the dominance of anions, i.e. carbonates, chloride, and sulfate. However, Soda chemical type as a group distinct from the carbonate type is rarely studied compared to other types. Our assessment of a representative data series of 220 lakes has shown that two major Soda types (Na-HCO_3_ and Na-HCO_3_-CO_3_) distinguished by the first dominant anions is insufficient to cover all subtypes with soda brine features. We have identified 22 soda subtypes in contrast with a mere number of eight saline subtypes, without soda brine character in their composition (Table 2). Similar results were reported in a previous study [27], where the authors used the same data and analysis that identified Na-HCO_3_ as a major type and 11 soda subtypes in a relatively small geographical area of the Carpathian basin. Such extensive diversity of ion composition (Fig 2) has been reported in numerous comprehensive papers using different major and subtype categories for Soda-type brines such as Na-CO_3_, Na-CO_3_-Cl-SO_4_, Na-HCO_3_-CO_3_, and Na-HCO_3_-CO_3_-Cl. These inconsistencies in the literature have led to confusion in identifying soda chemical-type waters worldwide.

According to Williams [22], the most common subtypes within the soda type were Na-HCO_3_, Na-HCO_3_-CO_3_, Na-HCO_3_-CO_3_-Cl, and Na-Cl-HCO_3_-CO_3_ (Table 1). As can be seen, chloride is the most common concomitant ion in soda subtypes, as it is in most saline waters (in the form of NaCl). However, some authors did not consider the importance of bicarbonate ions in the soda brine complex [21,22], although 6.8% of the waters in the current assessment over Eurasia have been found to be of Na-HCO_3_ type, and a considerably higher share of 81% was found in a previous regional study conducted in the Carpathian Basin [27].

Various worldwide lists of soda lakes have been reported in the literature, particularly by microbiologists in recent years [12,45–46]. However, not all of the lakes can actually be referred to as Soda type according to the definition we propose in this review. The comparison of water types by the Valyashko and dominant ion methods showed that lakes determined as both Soda (by Valyashko) and Saline type (by dominant ion) contain up to 2%−23e% of total carbonates (Table 5), which means that even a small concentration of soda brine can radically change the chemical environment [15]. In addition, certain lakes are characterized by high Na^+^ content varying from 85 to 99 e% but have lower Ca^2+^ and Mg^2+^ content (0−13 and 0−4 e%, respectively), which is in contrast with the Soda and Soda-Saline types of brines, where the amount of these ions can reach up to 29 and 44 e%. With regard to the assessed data, the Valyashko index (3) can be applied as a soda brine indicator, but using it as the only factor for the chemical classification of inland waters is insufficient, because it considers only the ratio of HCO_3_^–^ + CO_3_^2–^ and Ca^2+^ + Mg^2+^ ions.

Furthermore, the challenge of water classification can be further complicated if all detectable major ions are considered in the classification without the 25% equivalence limit, as done by some authors [29,32,35].

Grant [6] and Warren [7] argued that soda lake formation depends on low levels of dissolved calcium and magnesium among the cations as a result of carbonate precipitation. This process determines the chemical genesis of a soda brine environment. Our assessment clearly demonstrates that Mg^2+^ is often the second cation after Na^+^, and Mg^2+^ determines several subtypes within the Soda and Soda-Saline types of brines. Usually, magnesium does not form its own mineral, appearing only as an additional element during calcium mineral precipitation [31,47], if Mg-concentration is high enough (e.g., Salda Lake in Turkey). Moreover, magnesium can form very strong complexes with carbonate ions and organic substances (fulvic and humic acids), which can increase its accumulation in water [34].

Based on the results of the presented PCA ordination, our first hypothesis is generally supported. This hypothesis proposes the dominance of Na^+^ in the cation sequence, and HCO_3_^–^ + CO_3_^2–^ ions ranked first, second or third in the anion sequence (ordered by the rank of dominant ions), identified by a minimum of 25 equivalent percentage (e%), which has proved to be appropriate for distinguishing the soda chemical type from the diverse combination of major ions in saline waters (Figs 3 and 4).The identified “Soda” and “Soda-Saline” types of lakes and pans are named by conventionally used terminology coupled with a new and precise chemical definition.

Nowadays the scientific community agrees that the stage of Soda type water formation begins at the moment of water saturation with respect to Calcite (e.g. Lake Magadi) [48]. According to the Hardie & Eugster [21] evaporation path used in the majority of recent publications about geochemical evolution of solutions, the next stage of the closed basins evolution after Calcite precipitation is the whole removal of carbonates (HCO_3_^-^ and CO_3_^2-^) together with a depletion of Ca^2+^ in the solution and the formation of the Saline (Cl-Na-Mg or SO_4_-Na-Mg) type (e.g. Dead Sea or Aral Sea) [32]. The chemical characteristics of the Saline group are radically different from the Soda type: much lower pH, higher salinity values, saturation with respect to Thenardite and Halite, etc. However, there is a huge gap between these two types indicating the lack of studies in the area of the soda chemical types. Moreover, the question of a possibility of geochemical transformation from one chemical type to another (as an example, from Soda to Saline) still remains poorly studied. The implementing of Soda and Soda-Saline separation for closed basins classification allows to expand and strengthen the knowledge about the evolution processes of inland objects, especially in the area of soda formation mechanisms.

As can be seen from Fig 2 and Fig 5, the investigated three chemical types of waters (Soda, Soda-Saline and Saline) are very different in their chemical environment and geochemical features in terms of water-rock interaction, whereas the Soda-Saline stage of the chemical evolution combines the peculiar properties of both Soda and Saline (Cl or SO_4_) types. The Soda-Saline type solution has a lack of Ca^2+^ and the amount of HCO_3_^2-^+CO_3_^2-^ is constantly decreasing during carbonate precipitation, but, on the contrary, it may has higher Mg^2+^ concentration in comparison with the Soda types. Moreover, the amount of chloride and sulfate ions is constantly increasing during the evaporation processes that causes changes in transport features of many chemical elements [49]. For example, soda lakes and pans are usually associated with uranium accumulation [16; 50], since it creates strong complexes with carbonate ions, while a chloride dominated environment is perfect for lithium accumulation [51], as it has the same transport features as chloride or bromide ions. Taking into account that the characteristic environment of Soda-Saline types can combine the main features of two absolutely different chemical types, a deeper interpretation of that question needs more detailed chemical data including trace elements content that, unfortunately, were not presented in all of the data sources used in this study. However, the data obtained clearly indicate that the Soda-Saline type can be considered as a separate evolutionary stage between Soda and Saline types caused by intense evaporation of the Soda type solution. It should be noted, that in some cases this consistency may change due to the occurrence of extra sources of chloride or sulfate ions.

The Kruskal-Wallis test has demonstrated that pH values are significantly higher in Soda and Soda-Saline types (9.44 and 9.40) than in the Saline type of inland waters (8.50) (Fig 5). This result also allows us to confirm the validity of the identified “Soda” and “Soda-Saline” types derived from the PCA results. Besides, we reject the hypothesis that the pH range of Soda type is different from Saline waters because of the high overlapping coefficient (49%) of their pH density distribution (Fig 6). This is an important practical point because the term “alkaline” is often applied to waters merely on the basis of the alkaline pH value without considering chemical composition.

The well-known notion that salinity is lower and alkalinity is higher in soda lakes and pans than in chloride or sulfate ones has also been confirmed by the current study. Hammer [1] reassessed the pH values of hundreds of saline lakes around the world and found that in most of the saline lakes its value ranges from 7.5 to 9.5. However, in our dataset on Eurasian saline waters, pH can reach up to 10.4, which explains the 0.489 overlapping coefficient of pH distributions in the total range of observed values. The practical problem with pH is that it can significantly increase on a seasonal and daily basis if photosynthesic activity is high [52]. The measurement of pH usually takes place in the plant growth period and during the day when photosynthesic activity is intensive. This can lead to an overestimation of the pH value in temporary alkaline saline lakes, which can explain the weak correlation between salinity and pH (*r*^2^ = 0.330) when considering all types of inland saline waters in the dataset.

A good example of misclassification is Lake Khilganta (Southeastern Transbaikalia), which was identified by Namsaraev et al. [53] as a soda-saline lake, on the other hand, our analysis has shown that the lake is nor a soda neither a Soda-Saline type. Moreover, as can be seen from the data presented by the authors, its pH varied between 8.9 and 9.8 depending on dry or wet climatic periods. This example, together with our analysis, confirmed that pH alone is not a reliable factor for classifying a water body as soda chemical type. However, pH can be used as an additional criterion for the determination of an alkaline environment in inland saline systems if seasonal and diurnal pH data are available.

## Conclusions

This assessment across a large geographic scale and broad salinity range of 220 Eurasian saline inland surface waters resulted in a more precise identification and classification of soda chemical type of waters. For future research, we propose to use the “Soda” and “Soda-Saline” chemical types terminology for different kinds of brine, saline or brackish waters with a soda character if the ion composition meets the following fundamental criteria:

Soda: Na^+^ and HCO_3_^–^ + CO_3_^2–^ are the most dominant ions (> 25e%).

Soda-Saline: Na^+^ is ranked first among the dominant cations (> 25e%), but the sum of HCO_3_^–^ + CO_3_^2–^ is not of the most dominant component of the anions (> 25e%).

The application of the proposed terminology of “Soda” and “Soda-Saline” chemical types for soda brines can help reduce the level of misunderstanding between cross-disciplinary researches and also provide a clearer picture of chemical features. As described above, in some cases, saline lakes with Cl^−^ predominance can be incorrectly referred to as chloride or sulfate type, although they in fact have more in common with the soda-type features of the environment. Our extended classification of soda brines does not change the widely accepted classification of chemical water types, but it gives a precise vision to researchers from different fields for studying the environment in each particular case. Our analysis clearly demonstrates that pH measurement alone is not a reliable indicator for classifying the soda chemical type of inland saline surface waters.

As a practical consideration of our review, we are working on an online data input web tool for a worldwide database of the soda and saline chemical types of inland surface waters supported by Google Satellite raster data via OpenLayers plugin. This special tool requires the location of the sites by points or polygons and also allows uploading some basic geographical and limnological data. The most practical innovation of this web application will be the classification panel, where users can upload the concentration of major ions and automatically acquire the appropriate chemical type of the water according to the definitions described in this study. More information about this free service can be requested from the corresponding author.

## Supporting information

S1 Table Geographic and unpublished analytical data of additional sample series in the Eurasian region (sampling year was 2016). Footnotes Geographic Coordinate System: WGS 84.
(DOCX)
