# Supplementary material for: A review of the defining chemical properties of soda lakes and pans: An assessment on a large geographic scale of Eurasian inland saline surface waters
Source: PLoS One. 2018 Aug 20;13(8):e0202205. doi: 10.1371/journal.pone.0202205 (PMC6101393; doi:10.1371/journal.pone.0202205)
Supplement: S1 Table — Footnotes Geographic Coordinate System: WGS 84. (DOCX) [file pone.0202205.s001.docx]

**Supporting information**

**S1 Table** Geographic and unpublished analytical data of additional sample series in the Eurasian region (sampling year was 2016).

| **Latitude (N)** | **Longitude (E)** | **Country** | **Name** | **pH** | **Na^+^ (e%)** | **K^+^ (e%)** | **Ca**^2+^ **(e%)** | **Mg**^2+^ **(e%)** | **Cl**^–^ **(e%)** | **SO_4_^2–^ (e%)** | **HCO_3_^–^ (e%)** | **CO_3_^2–^ (e%)** |
| --- | --- | --- | --- | --- | --- | --- | --- | --- | --- | --- | --- | --- |
| 58.8205 | 48.3379 | Kazakhstan | Unknown | 8.29 | 62.36 | 0.44 | 21.35 | 15.85 | 28.51 | 25.36 | 46.13 | 0.00 |
| 59.5798 | 47.8805 | Kazakhstan | Unknown | 9.05 | 91.98 | 1.21 | 3.78 | 3.03 | 24.68 | 5.51 | 58.30 | 11.50 |
| 70.1005 | 53.0495 | Kazakhstan | Small Chebachie | 7.60 | 61.15 | 1.34 | 2.54 | 34.97 | 64.01 | 24.54 | 9.22 | 2.23 |
| 73.0055 | 53.8679 | Kazakhstan | Kishikaroy | 7.48 | 77.40 | 0.17 | 0.48 | 21.94 | 83.06 | 16.84 | 0.10 | 0.00 |
| 73.7690 | 53.4448 | Kazakhstan | Kyzylkak | 7.12 | 76.39 | 0.19 | 0.45 | 22.97 | 94.54 | 5.40 | 0.06 | 0.00 |
| 74.3616 | 52.7334 | Kazakhstan | Urpek | 7.24 | 69.53 | 0.23 | 6.89 | 23.34 | 73.99 | 25.58 | 0.43 | 0.00 |
| 75.3681 | 52.9261 | Kazakhstan | Shamantuz | 7.45 | 82.92 | 0.52 | 4.85 | 11.72 | 73.05 | 18.03 | 8.92 | 0.00 |
| 99.1608 | 45.5928 | Mongolia | Bodiltsagaan | 9.00 | 76.68 | 1.90 | 3.18 | 18.24 | 5.89 | 63.62 | 27.01 | 3.48 |
| 100.5761 | 45.0658 | Mongolia | Orog nuur | 9.37 | 77.72 | 2.77 | 0.85 | 18.65 | 38.97 | 22.68 | 30.48 | 7.87 |
| 100.7726 | 45.3248 | Mongolia | Kholboolj nuur | 9.10 | 77.95 | 6.40 | 3.50 | 12.15 | 29.96 | 18.77 | 44.75 | 6.53 |
| 101.9997 | 45.8027 | Mongolia | Unknown | 8.83 | 55.41 | 3.89 | 7.64 | 33.07 | 19.37 | 9.79 | 60.52 | 10.32 |
| 103.2995 | 46.6743 | Mongolia | Sangiin dalai | 8.82 | 49.30 | 2.71 | 10.74 | 37.25 | 15.53 | 37.94 | 40.40 | 6.12 |
| 108.2985 | 47.6295 | Mongolia | Gungaluut nuur | 9.23 | 50.53 | 3.35 | 1.74 | 44.37 | 8.27 | 40.46 | 45.19 | 6.08 |
| 112.1556 | 47.8286 | Mongolia | Gurmin Lake | 9.45 | 84.65 | 1.78 | 0.83 | 12.74 | 11.09 | 9.26 | 73.91 | 5.74 |
| 113.5906 | 49.3644 | Mongolia | Khairkhan | 9.43 | 92.45 | 5.36 | 0.05 | 2.13 | 38.21 | 25.17 | 30.63 | 6.00 |
| 114.6603 | 48.4414 | Mongolia | Unknown | 9.58 | 99.02 | 0.29 | 0.19 | 0.50 | 40.01 | 5.20 | 46.26 | 8.52 |
| 114.6645 | 49.5321 | Mongolia | Chukh Lake | 9.06 | 53.73 | 1.17 | 2.67 | 42.44 | 6.46 | 8.46 | 69.59 | 15.49 |
| 114.8690 | 49.1380 | Mongolia | Sumiin Lake | 8.95 | 68.15 | 2.41 | 0.98 | 28.46 | 7.02 | 62.91 | 24.63 | 5.44 |
| 115.5886 | 49.5181 | Mongolia | Khukh Lake | 9.23 | 85.16 | 0.66 | 1.04 | 13.13 | 31.97 | 11.47 | 46.01 | 10.55 |

Footnotes

Geographic Coordinate System: WGS 84.
